# Occurrence and impact of negative behaviour, including domestic violence and abuse, in men attending UK primary care health clinics: a cross-sectional survey

**DOI:** 10.1136/bmjopen-2014-007141

**Published:** 2015-05-11

**Authors:** M Hester, G Ferrari, S K Jones, E Williamson, L J Bacchus, T J Peters, G Feder

**Affiliations:** 1 School for Policy Studies, Centre for Gender and Violence Research, University of Bristol, Bristol, UK; 2 School of Social and Community Medicine, Centre for Academic Primary Care, University of Bristol, Bristol, UK; 3 Faculty of Public Health and Policy, Gender Violence & Health Centre, London School of Hygiene & Tropical Medicine, London, UK; 4 School of Clinical Sciences, University of Bristol, Bristol, UK

**Keywords:** PRIMARY CARE, prevalence, domestic violence and abuse, cross sectional survey, male patients

## Abstract

**Objective:**

To measure the experience and perpetration of negative behaviour, including domestic violence and abuse (DVA), and investigate its associations with health conditions and behaviours in men attending general practice.

**Design:**

Cross-sectional questionnaire-based study conducted between September 2010 and June 2011.

**Setting:**

16 general practices in the south west of England.

**Participants:**

Male patients aged 18 or older, attending alone, who could read and write English. A total of 1403 of eligible patients (58%) participated in the survey and 1368 (56%) completed the questions relevant to this paper. 97% of respondents reported they were heterosexual.

**Main outcome measures:**

Lifetime occurrence of negative behaviour consistent with DVA, perceived health impact of negative behaviours, associations with anxiety and depression symptoms, and cannabis use in the past 12 months and binge drinking.

**Results:**

22.7% (95% CI 20.2% to 24.9%) of men reported ever experiencing negative behaviour (feeling frightened, physically hurt, forced sex, ask permission) from a partner. All negative behaviours were associated with a twofold to threefold increased odds of anxiety and depression symptoms in men experiencing or perpetrating negative behaviours or both. 34.9% (95% CI 28.7% to 41.7%) of men who reported experiencing negative behaviour from a partner, and 30.8% (95% CI 23.7% to 37.8%) of men who perpetrated negative behaviours said they had been in a domestically violent or abusive relationship. No associations with problematic drinking were found; there was a weak association with cannabis use.

**Conclusions:**

DVA is experienced or perpetrated by a large minority of men presenting to general practice, and these men were more likely to have current symptoms of depression and anxiety. Presentation of anxiety or depression to clinicians may be an indicator of male experience or perpetration of DVA victimisation.

**Strengths and limitations of this study**

- This is the first survey of a European clinical population to measure prevalence of DVA experience and perpetration in male patients in primary care, and the largest such primary care study internationally.
- The study is unique in combining prevalence of experience and perpetration along with perceived impact and self-reported domestic violence and abuse (DVA) status. Unlike most population studies of men and DVA, it has no upper age limit.
- The study is cross-sectional and can only report associations. Men with higher education were over-represented in the sample, and gay and bisexual men were under-represented, compared with the UK population. While the preponderance of heterosexual men in the sample is a bias, the numbers of gay or bisexual men attending primary care practices is not known.
- Not all negative behaviours constitute DVA. Although a questionnaire cannot characterise the context of negative behaviours, we have measured frequency, severity and impact of negative behaviours, and included questions on whether men considered that they had been in a DVA relationship.
- It was not possible to calculate a true recruitment rate, as we do not know the absolute number of eligible men in the practice waiting rooms. However, given that men did not know that the questionnaire would include questions on abuse, the estimate of prevalence and impact may not have been affected. Men completed the survey while waiting to be called for their appointment and thus, had limited time to complete the survey and some were interrupted before completing it. All the data missing for this reason are, therefore, missing at random, justifying to some extent our imputation method.

## Introduction

The UK Government has defined domestic violence and abuse (DVA) as any incident or pattern of incidents of controlling, coercive or threatening behaviour, violence or abuse between those aged 16 or older who are or have been intimate partners regardless of gender or sexuality; this can include psychological, physical, sexual, financial or emotional abuse.^1^ However, estimates of DVA prevalence without taking into account context, severity and impact can be misleading.^2^ ^3^ Individuals may report that they have experienced or perpetrated one or more behaviours, but they may not actually experience or see these as harmful, or they may not perceive such behaviours as abusive, or may only define particular (usually physical) behaviours as abuse. DVA is a global health problem.^4^ Research on DVA has largely focused on heterosexual women as the largest victim group^5^ and there is a lack of research on men as victims or perpetrators of partner DVA.^3^ ^6^ ^7^

Female clinical populations generally have a higher prevalence of experience of DVA than the general population^8^ and this is probably the case for men experiencing or perpetrating DVA. In a US study of 712 men attending an emergency department, 20% disclosed abuse from a partner in the past year, 6% disclosed perpetration and 11% disclosed both.^9^ A UK study of 178 men in four general practices^10^ found a lifetime prevalence of 15% for men experiencing and 16% for men perpetrating DVA. In a Slovenian family practice sample of 323 men, 5% reported experience of psychological violence and 3% of men reported experience of physical violence from partners or family members in the previous 5 years.^11^

Men who experience or perpetrate DVA have an increased risk of post-traumatic syndrome, depression, suicide^7^ ^12^ ^13^ and substance misuse.^14^ Similarly, men with depressive or anxiety disorders are more likely to have experienced DVA^15^ and men with psychiatric disorders are more likely to report ever having been physically violent towards a partner.^16^ Such associations between adverse mental health and substance misuse with DVA may be stronger when men both experience and perpetrate DVA.^9^ However, it can be difficult to distinguish male victims from male perpetrators as the former may, for instance, present themselves as experiencing DVA when their partners have retaliated or defended themselves.^17–21^

Given the uncertainty about the prevalence of DVA experience or perpetration among men in primary care populations and its association with mental health problems, we conducted a study in English general practices. In this paper we report: (1) the occurrence of negative behaviours consistent with DVA (experienced from a partner and carried out towards a partner); (2) the perceived impact of abuse; (3) the association between exposure (experienced and carried out) with different types of DVA and mental health problems.

## Methods

### Study population

We selected a stratified random sample of general practices in south west England to reflect the profile of practice populations in England with respect to the proportion of patients from ethnic minorities (postcode level census data); levels of deprivation (index of multiple deprivation) and population density (city, town, village). Three practices declined to take part in the study; therefore, the next randomly selected practices from the relevant strata were recruited. From September 2010 to June 2011, we administered a questionnaire to unaccompanied male patients in the waiting rooms of 16 practices. Only those 18 years or older and able to read English were included, and men were not approached if they were unwell or appeared distressed.

### Variables

The questionnaire elicited demographic characteristics consistent with Crime Survey England and Wales categories: age in years, ethnicity, income, education and housing status.^22^ Current anxiety and depression were measured with the Hospital Anxiety and Depression Scale (HADS)^23^ by using a cut-off score of 8 for each subscale. We carried out sensitivity analyses with a cut-off score of 12 and a continuous measure for each of the subscales respectively. Alcohol use was measured with the Alcohol Use Disorders Identification Test (AUDIT),^24^ with a category of excessive drinking if the respondent usually drank more than 6 units of alcohol on a day when he drank. The cannabis use question was whether the respondent had used it in the past year.

The questionnaire included four questions about experience of behaviours that might be construed as DVA: (1) ever felt frightened of the behaviour of a partner; (2) ever needed to ask your partner's permission to work, go shopping, visit relatives or visit friends (beyond being considerate to and checking with your partner); (3) ever been slapped, hit, kicked or otherwise physically hurt; (4) and ever forced to have sex or made to engage in any sexual activity when you did not want to. This was followed by questions about their relationship with the perpetrator, frequency and escalation of the experience, and perceived impact of the behaviours based on the Comparing Heterosexual and Same Sex Abuse in Relationships (COHSAR) survey.^3^ The subsequent questions were about perpetration of any of the four negative behaviours towards a current or former partner, whether this had occurred in the past 12 months, and whether they felt these behaviours had an effect on their partner. An additional question was whether the participant had experienced any of the negative behaviours from an adult family member other than a partner. Two questions were whether they were currently or had been in a relationship that could be described as ‘domestically violent or abusive’. In this paper, we differentiate between the responses to the behavioural questions and the self-perceived DVA with the term ‘negative behaviour’ to denote the former and ‘DVA’ the latter.

The questionnaire booklet contained a detachable information sheet with contact details of support services and national help lines. Respondents were also encouraged to talk to the researcher who recruited them if the survey raised issues that caused them concern.

### Study size

The sample size was driven by our aim of investigating associations between experience or perpetration of DVA and demographic variables or mental health problems. Assuming a conservative background prevalence of 10% for depression among men attending general practice, a sample size of 1400 respondents from 14 to 16 practices would have 88% power to detect a 15 percentage point difference between men who have experienced or perpetrated negative behaviours, and men who have not done so with regard to any demographic or health measure. This calculation used a two-sided 5% significance level and assumed a design effect of 2 from clustering within practices, based on a cluster size of 100 and an ICC of 0.01. This sample size also allowed us to estimate a prevalence of perpetration of 10% with a 2.2% margin of error after adjusting for clustering.

### Statistical analysis

Data were entered from the paper questionnaire into an Access database. Only individuals who responded to all four questions on negative behaviours were included; two respondents were excluded because they were younger than 18. All data were analysed in Stata V.12.1.^25^ We compared proportions of participants experiencing or perpetrating negative behaviours with the proportion of men describing their current or previous relationship as domestically violent or abusive. To investigate whether experience or perpetration of negative behaviours was associated with mental health problems or substance abuse, we performed logistic regressions for the latter variables as outcomes controlling for age, income, maximum level of education and house ownership. In the analysis, we also accounted for the stratification and clustering in the study design. The variables included in the models were identified a priori*,* based on previous evidence of associations between DVA, socioeconomic status, drug use and health outcomes.^9^ ^12^ ^13^

The main analysis is based on complete cases, using the binary versions of the mental health outcomes with a cut-off score of 8. These were then repeated for the continuous score and an alternative cut-off of 12. For another sensitivity analysis, we repeated the main analysis using imputed missing data with chained equations (mice) and then recalculated our estimates on the imputed data sets making the assumption that the data are missing at random^26^ and thus, generated 100 complete data sets.

### Medical records

Consent was sought from participants for access to their medical records. Electronic records for men who consented were accessed in the practices by two researchers who extracted information from any documentation of DVA. The same 12-month period was used for survey and medical records.

## Results

### Sociodemographic characteristics

Of the 2431 eligible men invited to complete the survey, 1403 (58%) consented and 1368 (56%) completed the questions relevant to this paper. Recruitment rates in practice waiting rooms varied between 35% and 72%.^27^ Participants were aged between 18 and 90 years (mean 49 years); 68.2% (95% CI 65.4% to 71.2%) had a national vocational, advanced or higher qualification; and 97.1% (95% CI 96.2% to 98%) were heterosexual. Table 1 reports the sociodemographic profile of all respondents with additional subdivision into men who reported experiencing or perpetrating negative behaviours (or both), and those who were not involved in such behaviours. The groups are broadly similar although those without any history of negative behaviours are more likely to be in paid employment and to be living with a partner.

**Table 1:** Sociodemographic characteristics of the participants*

	Victims only (N=162)			Perps only (N=93)			Both V and P (N=117)		
	n	Per cent	95% CI	n	Per cent	95% CI	n	Per cent	95% CI
Mean age SD Range N	44 16 (19, 90) 159			46 16 (18, 80) 85			43 15 (19, 85) 111		
White	151	94.4	(89.8% to 97.8%)	82	92.1	(84.0% to 98.5%)	109	95.6	(90.9% to 99.6%)
Mixed	3	1.9	(0.0% to 5.2%)	4	4.5	(−0.2% to 11.5%)	0		
Asian or Asian British	5	3.1	(0.1% to 6.8%)	1	1.1	(−1.0% to 4.5%)	1	0.9	(−0.7% to 4.2%)
Black or Black British	1	0.6	(−0.4% to 2.8%)	2	2.2	(−0.9% to 7.2%)	3	2.6	(−0.1% to 6.6%)
Chinese or other	5	5.6	(1.2 to 11.9)	0			1	0.9	(−0.5% to 3.1%)
Currently has partner	112	70.4	(61.4% to 78.2%)	69	74.7	(63.7% to 84.6%)	73	65.2	(52.8% to 75.6%)
Currently has no partner	47	29.6	(21.3% to 38.0%)	23	25.3	(15.4% to 36.7%)	39	34.8	(23.1% to 46.7%)
Lives with this partner	84	77.1	(67.3% to 86.1%)	52	76.5	(61.9% to 91.0%)	55	77.5	(61.5% to 91.0%)
Does not live with this partner	25	22.9	(14.2% to 32.9%)	16	23.5	(10.0% to 37.6%)	16	22.5	(8.0% to 37.7%)
Is a parent	91	56.9	(45.9% to 66.0%)	54	59.3	(45.8% to 70.9%)	80	69.0	(57.7% to 80.6%)
Is not a parent	69	43.1	(32.8% to 53.2%)	37	40.7	(28.2% to 54.4%)	36	31.0	(19.5% to 42.3%)
In paid employment	107	78.7	(70.0% to 86.5%)	55	75.3	(61.4% to 87.4%)	64	64.6	(52.2% to 76.5%)
Not in paid employment	29	21.3	(13.9% to 30.1%)	18	24.7	(12.1% to 40.1%)	35	35.4	(24.9% to 48.1%)
Up to £10 000	39	28.9	(20.7% to 38.7%)	24	32.0	(18.1% to 47.4%)	26	29.2	(15.9% to 41.3%)
£11 000–£20 000	44	32.6	(24.1% to 41.8%)	20	26.7	(12.5% to 42.2%)	30	33.7	(21.3% to 46.5%)
£21 000–£30 000	33	24.4	(17.1% to 32.3%)	16	21.3	(9.4% to 33.6%)	15	16.9	(8.3% to 26.5%)
£31 000–£40 000	10	7.4	(2.8% to 13.0%)	7	9.3	(0.6% to 19.7%)	10	11.2	(3.8% to 21.3%)
£41 000–£50 000	5	3.7	(0.4% to 7.8%)	5	6.7	(−0.8% to 15.7%)	2	2.2	(−0.7% to 6.4%)
£51 000–£60 000	1	0.7	(−0.3% to 3.1%)	1	1.3	(−1.2% to 5.4%)	3	3.4	(−0.9% to 9.4%)
More than £60 000	3	2.2	(−0.3% to 5.6%)	2	2.7	(−2.1% to 9.1%)	3	3.4	(−0.3% to 8.6%)
No education	15	9.8	(5.2% to 15.3%)	12	13.3	(5.5% to 23.2%)	16	14.3	(7.4% to 23.2%)
GCSE/O Level	22	14.4	(8.0% to 20.9%)	15	16.7	(6.5% to 28.0%)	18	16.1	(8.2% to 24.5%)
A Level	24	15.7	(8.5% to 23.6%)	14	15.6	(6.6% to 24.0%)	20	17.9	(9.2% to 28.0%)
NVQ	24	15.7	(9.3% to 21.9%)	20	22.2	(10.7% to 35.2%)	23	20.5	(12.1% to 30.0%)
Professional Qualification	22	14.4	(8.2% to 21.5%)	14	15.6	(6.7% to 26.2%)	13	11.6	(4.4% to 20.4%)
Undergraduate Degree	28	18.3	(11.6% to 25.8%)	9	10.0	(2.9% to 18.4%)	15	13.4	(4.5% to 22.5%)
Postgraduate Degree	18	11.8	(6.3% to 17.8%)	6	6.7	(0.8% to 13.9%)	7	6.3	(1.1% to 13.0%)
Private owned	68	43.6	(33.4% to 52.8%)	45	48.4	(34.5% to 61.6%)	44	38.6	(27.5% to 50.9%)
Private rented	57	36.5	(25.7% to 46.4%)	24	25.8	(14.4% to 37.0%)	33	28.9	(18.0% to 39.1%)
Council housing	25	16.0	(9.3% to 23.5%)	18	19.4	(9.7% to 29.6%)	27	23.7	(13.9% to 33.4%)
Other	6	3.8	(0.7% to 7.7%)	6	6.5	(0.3% to 13.8%)	10	8.8	(3.2% to 15.2%)

	Neither victims nor perpetrators (N=914)			Whole sample (N=1368)		
	n	Per cent	95% CI	n	Per cent	95% CI
Mean age SD Range N	50 19 (18, 90) 882			49 18 (18, 90) 1308		
White	854	95.7	(94.3% to 97.1%)	1267	95.1	(93.8% to 96.3%)
Mixed	12	1.3	(0.6% to 2.2%)	23	1.7	(1.1% to 2.5%)
Asian or Asian British	7	0.8	(0.3% to 1.5%)	14	1.1	(0.6% to 1.7%)
Black or Black British	14	1.6	(0.8% to 2.6%)	22	1.7	(0.9% to 2.4%)
Chinese or other	0			6	4.5	(1.5 to 9.4)
Currently has partner	678	75.8	(72.7% to 78.7%)	980	73.6	(71.1% to 76.5%)
Currently has no partner	216	24.2	(21.2% to 27.0%)	352	26.4	(24.0% to 29.0%)
Lives with this partner	586	87.5	(84.5% to 90.5%)	819	84.8	(81.6% to 87.3%)
Does not live with this partner	84	12.5	(9.5% to 16.0%)	147	15.2	(12.6% to 18.2%)
Is a parent	579	64.3	(60.6% to 68.1%)	861	63.9	(60.5% to 67.3%)
Is not a parent	322	35.7	(31.7% to 39.6%)	487	36.1	(33.1% to 39.7%)
In paid employment	521	82.3	(79.3% to 85.5%)	776	78.9	(75.7% to 81.6%)
Not in paid employment	112	17.7	(14.5% to 20.8%)	208	21.1	(18.2% to 24.1%)
Up to £10 000	174	24.2	(20.8% to 27.6%)	282	26.3	(23.7% to 29.3%)
£11 000–£20 000	227	31.6	(28.2% to 35.0%)	341	31.8	(29.1% to 34.9%)
£21 000–£30 000	158	22.0	(19.1% to 24.8%)	235	21.9	(19.9% to 24.7%)
£31 000–£40 000	81	11.3	(9.0% to 13.9%)	108	10.1	(8.3% to 12.1%)
£41 000–£50 000	46	6.4	(4.6% to 8.3%)	60	5.6	(4.2% to 6.9%)
£51 000–£60 000	14	1.9	(1.0% to 3.2%)	19	1.8	(1.0% to 2.6%)
More than £60 000	19	2.6	(1.5% to 4.0%)	27	2.5	(1.6% to 3.5%)
No education	125	14.5	(12.1% to 17.1%)	191	14.8	(12.9% to 17.3%)
GCSE/O Level	149	17.3	(14.7% to 20.4%)	218	16.9	(14.8% to 19.0%)
A Level	82	9.5	(7.4% to 11.6%)	143	11.1	(9.3% to 12.8%)
NVQ	127	14.8	(11.9% to 17.5%)	202	15.7	(13.5% to 17.9%)
Professional Qualification	162	18.8	(16.4% to 22.1%)	223	17.3	(15.2% to 19.6%)
Undergraduate Degree	141	16.4	(13.5% to 19.2%)	203	15.8	(13.4% to 18.1%)
Postgraduate Degree	75	8.7	(6.7% to 10.6%)	108	8.4	(6.9% to 10.2%)
Private owned	595	66.0	(62.6% to 70.1%)	799	59.4	(56.4% to 63.0%)
Private rented	165	18.3	(15.0% to 21.4%)	290	21.6	(18.9% to 24.4%)
council housing	86	9.5	(7.6% to 11.7%)	173	12.9	(11.0% to 14.9%)
Other	55	6.1	(4.6% to 7.7%)	82	6.1	(4.9% to 7.4%)

*All CIs calculated as per Ng *et al,* 2013.

GCSE, General Certificate of Secondary Education; NVQ, National Vocational Qualifications.

Table 2 reports the experience and perpetration of negative behaviours from and against a partner for three groups: those men who only experienced, those who only perpetrated and those who both experienced and perpetrated the behaviour, as well as the reporting of any negative behaviours in the past 12 months.

**Table 2:** Reported negative behaviours

	Only from a partner N=162		Only towards a partner N=93		Both from and toward a partner N=117	
	N	Per cent	N	Per cent	N	Per cent
Ever frightened	97	59.9 (51.4 to 68.9)	86	92.5 (82.5 to 98.4)	62	53.0 (42.3 to 63.7)
Ever had to ask permission	66	40.7 (32.4 to 49.5)	3	3.2 (−0.4 to 9.4)	19	16.2 (8.7 to 25.4)
Ever hurt	80	49.4 (40.2 to 57.6)	18	19.8 (10.4 to 29.5)	35	29.9 (20.1 to 40.5)
Ever forced to have sex	17	10.5 (5.0 to 15.9)	4	4.3 (−0.4 to 9.8)	2	1.7 (−0.3 to 5.1)
Any the past 12 months	43	28.5 (20.0 to 37.3)	23	28.8 (16.3 to 43.9)	28	25.5 (16.0 to 35.5)

All CIs calculated as per Ng *et al,* 2013.

### Prevalence and frequency of negative behaviours experienced from a partner

While most negative behaviours were from partners, 28 of 1368 (2.1%; 95% CI 1.3 to 2.9) experienced them from an adult family member: 4 from their parents, 8 from their sons or daughters, 18 from other family or in-laws and 2 from both. Twenty-seven out of 1367 men (2%; 95% CI 1.2 to 2.9) reported perpetrating a negative behaviour against a family member: 3 against a brother or a parent, 12 against adult children and 12 against other family members or in-laws.

A total of 309 men (22.7% of respondents, 95% CI 20.2% to 24.9%) reported having at some time experienced at least one of the negative behaviours from a partner, including men who also reported perpetration. Of the total, 107 (41.2%, 95% CI 34.3% to 47.8%) said the behaviour had occurred only once, and 66 (25.4%, 95% CI 19.8% to 31.6%) that it had occurred more than once and for over a year. The behaviour stayed the same for 154 men (60.9%, 95% CI 54.5 to 66.8), got worse for 60 (23.7%, 95% CI 18.1% to 29.7%) and worsened and became more frequent for 15 men (5.9%, 95% CI 3.1% to 9.5%).

One hundred and two respondents (7.6%, 95% CI 6.2% to 9.1%) reported experiencing any negative behaviours in the past 12 months. Only eight of all the 1368 respondents reported being in a current DVA relationship; we, therefore, omitted this variable from further analysis.

### Prevalence of negative behaviours carried out towards a partner

Combining participants who reported only perpetrating negative behaviours on a partner with those perpetrating and experiencing negative behaviour, 212 of 1294 male respondents (16.4%, 95% CI 14.3% to 18.5%) reported perpetration of negative behaviours against a partner at least once. Fifty-eight of 1283 male respondents (4.5%, 95% CI 3.5% to 5.8%) reported perpetrating any negative behaviours in the past 12 month.

### Distributions of negative behaviours experienced, carried out or both

Being frightened or causing fright was the most common negative behaviour experienced or perpetrated. Overall 106/1323 (8%, 95% CI 6.6%, 9.4%) men reported both exposures.

### Reported negative behaviours and perceptions of being in a DVA relationship

Only 34.9% (95% CI 28.7% to 41.7%) of men who reported experiencing negative behaviours said they had ever been in a DVA relationship. Reporting one's relationships as DVA was positively associated with the frequency (p<0.001) and duration (p<0.001) of the behaviour, without controlling for sociodemographic characteristics; experiencing multiple forms of negative behaviours was also associated with ever having been in a DVA relationship (p<0.001). In total 30.8% (95% CI 23.7% to 37.8%) of men who perpetrated negative behaviours said they had ever been in a DVA relationship and were more likely to report this if they perpetrated more than one type of negative behaviour (p<0.001). As indicated, only eight of the 1368 respondents reported being in a *current* DVA relationship and this variable was omitted from further analysis.

### Health problems associated with negative behaviour experienced from a partner

Table 3 shows the perceived effects of negative behaviours experienced from a partner. The most frequently reported effect is feeling anxious or depressed. Two-thirds of victims reported the behaviour had some effect on their lives (N=173; 67.1%; 95% CI 60.9% to 72.8%).

**Table 3:** Perceived impacts of negative behaviour from a partner

Impact	N	Per cent	95% CI
It damaged my physical health	28/201	13.9	(9.0 to 19.3)
It made me feel anxious or depressed	132/201	65.7	(58.3 to 73.1)
It made me drink more alcohol/take more drugs	62/201	30.8	(23.6 to 38.8)
It affected my work or studies	68/201	33.8	(26.1 to 41.3)
It affected my relationship with my children	41/201	20.4	(13.1 to 27.0)
Other effects	40/203	19.7	(13.9 to 25.9)

All CIs calculated as per Ng et al, 2013.

Men who experienced negative behaviours were in most cases at least twice as likely to report symptoms of anxiety and depression as men who had not experienced negative behaviours, with the odds of symptoms of anxiety and depression especially high for men who experienced negative behaviours in the past year (table 4).

**Table 4:** Negative behaviour from partner, anxiety and depression

	Anxiety binary				Depression binary			
	ORs	95% CI	p Value	Sample	ORs	95% CI	p Value	Sample
Ever frightened by partner	2.7	(1.9 to 3.9)	<0.001	961	1.8	(1.2 to 2.7)	0.0026	960
Ever had to ask permission from partner	2.2	(1.6 to 3.1)	<0.001	961	2.6	(1.6 to 4.2)	<0.001	960
Ever physically hurt by partner	2.5	(1.9 to 3.2)	<0.001	961	1.9	(1.2 to 3.0)	0.0091	960
Suffered negative behaviours in past year	3.7	(1.8 to 7.7)	<0.001	948	2.8	(1. 6 to 5.0)	<0.001	947
In abusive relationship in the past	3.2	(2.0 to 5.0)	<0.001	925	2.0	(1.3 to 3.3)	0.0034	924

The table reports ORs from logistic regressions for the mental health outcomes that include each of the listed experiences of NBs in turn, and controls for age, income, education, house ownership and sampling stratification. All CIs calculated as per Ng et al, 2013.

Men who were ever frightened by their partner had greater odds of smoking cannabis in the past year (OR 1.9, 95% CI 1.0 to 3.6), but there was no evidence of associations with other negative behaviours. We also found no association with excessive drinking despite one-third of the victims who reported on the effect of exposure saying it impacted on their own drinking behaviour.

### Health problems associated with negative behaviours carried out towards a partner

One-third of the men who perpetrated (63, 35.4%) thought their perpetration of negative behaviours had had a negative impact on their partners (95% CI 28% to 43.6%). The mental health of men perpetrating negative behaviours was similar to those experiencing negative behaviours, with worse mental health scores for men who reported having been in a DVA relationship at some point in the past (table 5). Men who used some form of negative behaviour against their partners were three to five times more likely to report symptoms of anxiety than non-perpetrators. Men who perpetrated a negative behaviour in the past year were almost five times more likely than non-perpetrators to report symptoms of anxiety (OR 4.6; 95% CI 2.2 to 9.7). Perpetration of negative behaviours was clearly positively associated with symptoms of depression except for physically hurting a partner, where the evidence was marginal.

**Table 5:** Negative behaviour towards partner, anxiety and depression

	Anxiety binary				Depression binary			
	ORs	95% CI	p Value	Sample	ORs	95% CI	p Value	Sample
Ever frightened partner	3.0	(2.1 to 4.2)	<0.001	927	2.9	(1.9 to 4.5)	<0.001	926
Ever wanted partner to ask for permission	4.7	(1.8 to 11.8)	0.0011	927	3.1	(1.5 to 6.4)	0.0018	926
Ever physically hurt partner	3.8	(1.8 to 8.1)	<0.001	927	1.9	(0.9 to 4.1)	0.089	926
Perpetrated negative behaviours in past year	4.6	(2.2 to 9.7)	<0.001	913	3.0	(1.4 to 6.2)	0.0033	912
In abusive relationship in the past	3.3	(2.1 to 5.2)	<0.001	903	2.1	(1.3 to 3.4)	0.0020	902

The table reports ORs from logistic regressions for the mental health outcomes that include each of the listed experiences of NBs in turn, and controls for age, income, education, house ownership and sampling stratification. All CIs calculated as per Ng et al, 2013.

### Sensitivity analysis—experience of negative behaviours

Analysis using a higher threshold for symptoms of both depression and anxiety gave results consistent with the analysis using lower thresholds. Point estimates are either essentially unchanged or between 30% and 40% smaller for anxiety, with less precision. ORs for negative behaviours and depression remain positive, but are reduced in some instances.

Analysis on the data sets we generated by multiple imputation using chained equations shows persistent positive associations between negative behaviours and poor mental health. For anxiety, the magnitude of association decreases for most negative behaviours. Results for depression are mixed: while still indicative of a positive association, the odds of being depressed if one experiences negative behaviour decreases in the imputed analyses by 9% on average for experience of any behaviour and having to ask for permission and increases by 31% on average for being physically hurt, having to ask for permission and for having been in a relationship characterised as DVA in the past.

### Sensitivity analyses—perpetration of negative behaviours

Analysis using the 12+ thresholds for both anxiety and depression scales resulted in positive estimates for the association with negative behaviours, but these were smaller than in the main analysis. For depression, all associations with perpetration of negative behaviours are non-significant. Consistent with the main analysis, the continuous HADS measures record higher average scores for perpetrators compared with non-perpetrators.

### Audit of medical records

Of the sample of 1368 men, 491 consented to having their medical records reviewed. During the same 12-month period, only two of 434 men (0.5%) had explicit partner victimisation or perpetration stated on their medical record, compared to 32 men (7.4%, 95% CI 4.6% to 10.3%) reporting this in the survey.

## Discussion

While a large majority of men reported never having been in a relationship that could be described as ‘domestically violent or abusive’, nearly a quarter reported ever having experienced a negative behaviour from a partner, and one-sixth reported perpetrating some form of violence against a partner. Men who reported experiencing negative behaviours were about twice as likely to report symptoms of depression as men who had not experienced such behaviours, and men who reported perpetrating negative behaviours were even more likely to report symptoms of anxiety and depression compared to men who did not perpetrate. There were no associations between problematic drinking patterns and negative behaviours, and there was no consistent association with cannabis use.

### Strengths and weaknesses

There is a lack of epidemiological research on men and DVA. This is the first survey of a European clinical population to measure prevalence of negative behaviour and DVA experience and perpetration for male patients, and the largest such primary care study internationally. It is unique in combining prevalence of experience and perpetration along with perceived impact and self-reported DVA status. Unlike most population studies of men and DVA, it has no upper age limit.^28^

The study is cross-sectional and can only report associations. Not all negative behaviours constitute DVA. It is difficult to distinguish between coercive control or intimate partner terrorism and negative behaviours in the context of situational couple violence, especially when women and men may not identify persistent, highly abusive behaviours as DVA.^29^ Although a questionnaire cannot characterise the context of negative behaviours, we have measured frequency, severity and impact of negative behaviours and included questions on whether men considered that they had been in a DVA relationship.

It was not possible to calculate a true recruitment rate, as we do not know the absolute number of eligible men in the practice waiting rooms. The 42% of men declining to participate introduces potential bias. Given that men did not know that the questionnaire would include questions on abuse, the estimate of prevalence and impact may not have been affected. Men completed the survey while waiting to be called for their appointment. This means that they had limited time to complete the survey and some were interrupted before completing it. All the data missing for this reason are, therefore, missing at random, justifying to some extent our imputation method. Men with higher education were over-represented in the sample, and gay and bisexual men were under-represented, compared with the UK population.

### Relation to other studies

Nearly one in four men in our survey reported a lifetime experience of negative behaviours consistent with DVA, which is more than twice the level reported by men in the UK population;^28^ this concords with findings in women showing increased prevalence in clinical populations.^8^ A US emergency department sample of largely African-American men given similar questions reported a past year DVA experience of 20% for current relationships,^9^ whereas men in our study reported only 5.2% negative behaviours in the past year, and mainly in a *previous* relationship. Unlike the national Crime Survey England and Wales (CSEW) data, where lower socioeconomic status groups had higher risk for experiencing DVA,^30^ our study found no consistent evidence of an association with socioeconomic status.

With regard to perpetration, 16% of men in our study reported perpetrating negative behaviours at some time, which is the same as in a small UK study.^10^ A US study found 6% perpetration in the past year, nearly double that reported in our study (3.2%).^9^ The CSEW does not ask about perpetration and thus, comparison with UK population prevalence data is not possible. The lower prevalence of reported perpetration compared with experience of negative behaviours in our sample may be a result of men under-reporting perpetration.^31^ ^32^ General population surveys report that women experience more emotional than physical abuse from male partners. The men in our research fitted this heterosexual pattern in that they were more than twice as likely to mention perpetrating frightening their partner rather than being physically violent.

While nearly a third of the men had experienced or perpetrated a negative behaviour from or towards a partner (or both), about two-thirds of these men did not think their relationships had ever been characterised by DVA. Men were more likely to report that they had been in a DVA relationship if they had experienced or perpetrated physical violence, and when there had been more than one form of negative behaviour. Despite the increasingly wide definition used by the UK government that we cite in our introduction, in the popular imagination DVA often conjures up a public story related to the heterosexual experience that also emphasises physical violence.^33^ The association between increased frequency or multiple forms of negative behaviour and increased likelihood of self-identification of DVA has been found in other studies, both for women^34^ and for men.^3^ The link between self-perceived DVA and such combinations are also consistent with the idea of DVA relationships as patterns of coercive control that span across the emotional, physical and sexual dimensions, rather than being confined to one dimension only.^1^ ^35^

### Mental health problems

We found a strong association between negative behaviours consistent with DVA and mental health problems in men, which has also been reported in population studies.^16^ ^36^ In an emergency department setting, Rhodes *et al*^9^ found that the likelihood of depression, PTSD, and suicidal ideation was greatest for men who reported experience *and* perpetration of intimate partner violence, which was not the case for this primary care population. Men who reported experiencing or perpetrating negative behaviours had a greater likelihood of anxiety and depressive symptoms than men who did not report negative behaviours, whether or not they reported that that they had been in a DVA relationship. The odds of reporting symptoms of anxiety and depression were generally higher for men who perpetrated negative behaviours than for those who experienced them. The strongest associations were for men who perpetrated a negative behaviour in the past year. In their systematic review of patients in psychiatric services, Oram *et al*^16^ also found an association between perpetration with anxiety and depressive disorders.

Surveys of general and clinical populations,^37^ mostly in the USA, identify alcohol use as a major risk factor associated with DVA perpetration, although the Crime Survey for England and Wales did not find that alcohol was independently associated with DVA.^30^ In our study, we found no association between negative behaviours and measures of alcohol abuse, although almost a third of men who had experienced negative behaviours from a partner reported that this had resulted in drinking more alcohol or using more illegal drugs. Cannabis use was not associated with either experience or perpetration of negative behaviours. Regarding illicit drug use, there are conflicting findings from a small number of other studies. The lack of association in our study between cannabis use in the past year and negative behaviours is consistent with Cunradi *et al*^38^ general population-based study in the USA that found no association between drug abuse and perpetration of intimate partner violence (IPV).

### Meaning of the study

The prevalence of negative behaviours towards a partner or adult family member (whether or not this is perceived as DVA) either experienced or perpetrated by men attending general practices and their association with mental health problems requires a response from services. Men who experience or perpetrate DVA see doctors as their main source of professional help, though most of them do not explicitly seek it. In the Canadian 2004 General Social Survey, men who reported IPV mostly reported formal help seeking via health professionals.^39^ Interviews with men attending DVA perpetrator programmes in the UK suggest that male perpetrators are likely to seek help from general practice,^40^ although in the Crime Survey for England and Wales, only 4% of men experiencing DVA reported that they would tell a health professional about the abuse. Nevertheless, the majority of men do not object to being asked about negative behaviours in the context of mental health or other problems associated with abuse.^10^ ^41^

If the male experience or perpetration of current or past DVA is not recognised by clinicians, their management of the associated mental health problems will be inadequate. In the UK, this has been recognised for women experiencing DVA, resulting in training and support programmes in primary care to improve identification of DVA survivors and referral to specialist DVA services.^42^ Our findings of a virtual absence of recording of DVA in records of male patients show that we need a parallel response to male patients with a history of experiencing or perpetrating DVA, and possibly specialist DVA services for men to which clinicians can refer to after a disclosure.

We need a better understanding of the nature and health effects of DVA experienced and perpetrated by men. While our study measured the prevalence of negative behaviours consistent with a broad definition of DVA, we were not able to determine what proportion of men were experiencing coercive control and the level of DVA severity that can be classified as ‘intimate terrorism’,^29^ ^35^ and whether these men suffer more severe health consequences. Further research also needs to focus on the development and evaluation of interventions for male survivors and perpetrators of DVA, especially since there is only weak evidence for the effectiveness of current programmes.^43^
